# Supplementary figures and images for: Growth, Proliferation and Metastasis of Prostate Cancer Cells Is Blocked by Low-Dose Curcumin in Combination with Light Irradiation
Source: Int J Mol Sci. 2021 Sep 15;22(18):9966. doi: 10.3390/ijms22189966 (PMC8469895; doi:10.3390/ijms22189966)

S1

Protein expression

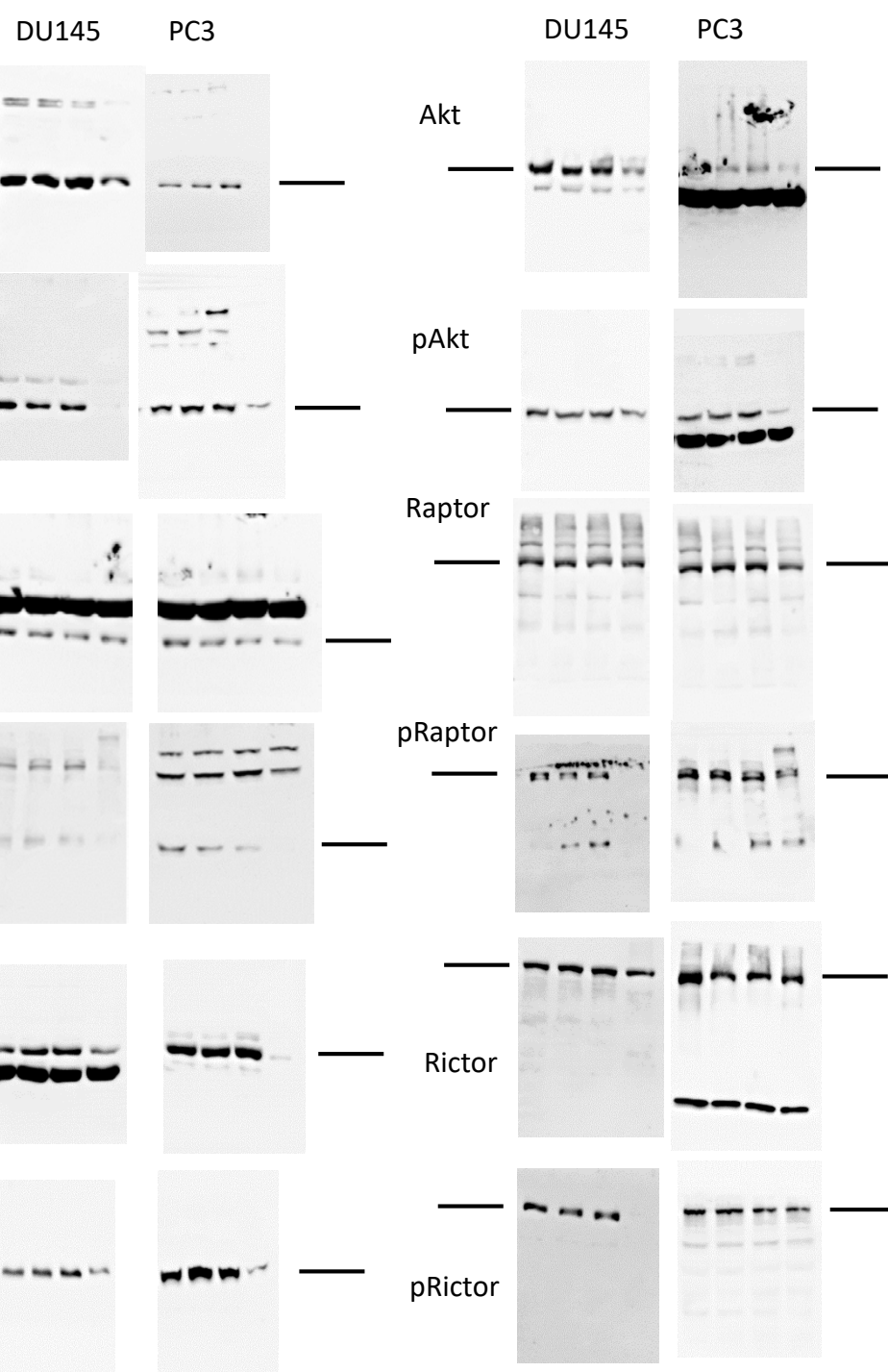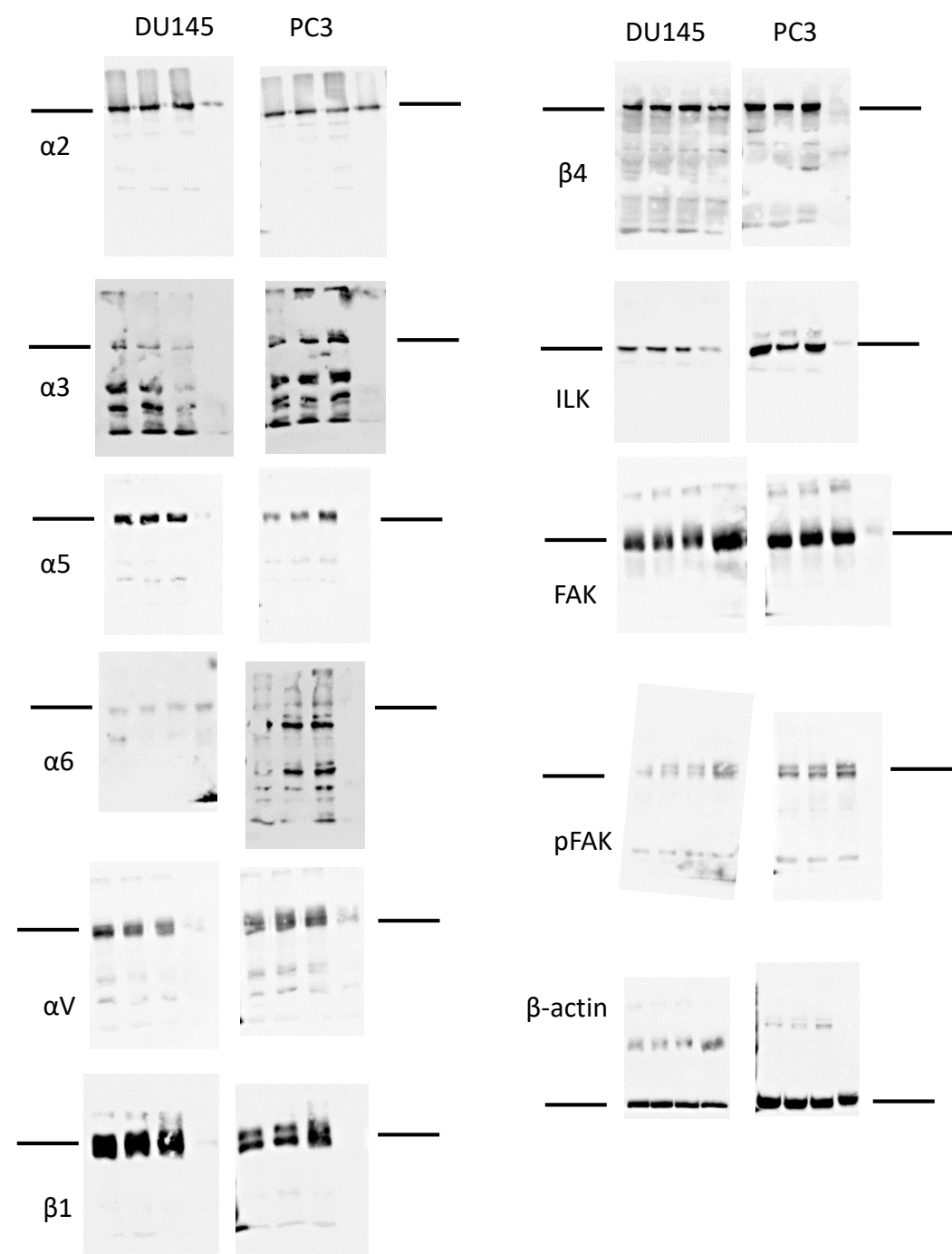

Supplement: Supplementary file 1 [file ijms-22-09966-s001.zip › ijms-1327106-supplementary.pdf]
